# Migraine and Stroke: In Search of Shared Pathways, Mechanisms, and Risk Factors

**DOI:** 10.7759/cureus.20202

**Published:** 2021-12-06

**Authors:** Mohammad Hassan, Rishab Belavadi, Sri Vallabh Reddy Gudigopuram, Ciri C Raguthu, Harini Gajjela, Iljena Kela, Chandra L Kakarala, Srimy Modi, Ibrahim Sange

**Affiliations:** 1 Internal Medicine, Mohi-ud-Din Islamic Medical College, Mirpur, PAK; 2 Surgery, Jawaharlal Institute of Postgraduate Medical Education and Research, Pondicherry, IND; 3 Research, Our Lady of Fatima University College of Medicine, Hyderabad, IND; 4 Research, Tianjin Medical University, Tianjin, CHN; 5 Research, Our Lady of Fatima University College of Medicine, Valenzuela, PHL; 6 Family Medicine, Jagiellonian University Medical College, Krakow, POL; 7 Internal Medicine, Jawaharlal Institute of Postgraduate Medical Education and Research, Pondicherry, IND; 8 Research, K. J. Somaiya Medical College, Mumbai, IND; 9 Research, California Institute of Behavioral Neurosciences & Psychology, Fairfield, USA

**Keywords:** migraine-associated vasospasm, cortical spreading depression, migraine-related stroke, hemorrhagic stroke, ischemic stroke, migraine with aura, stroke, migraine

## Abstract

Migraines are one of the emerging causes of disabilities experienced worldwide, and strokes are the second leading cause of death globally. Migraines with aura have been reported to be associated with a higher risk of ischemic strokes, whereas hemorrhagic strokes are more closely associated with migraines without aura, possible mechanisms that link migraines to strokes. These can be categorized into vascular mechanisms such as vasospasm, endothelial and platelet dysfunction, and alteration in the vessel wall seen in migraineurs, further perpetrated by vascular risk factors such as hypertension and hyperlipidemias. Cerebral hypoperfusion that occurs in migraines can cause an electrical aberrance, leading to a phenomenon known as “spreading depression” which can contribute to strokes. In this review, we discuss bloodstream elevation in procoagulants such as antiphospholipid antibodies, homocysteine, von Willebrand factor, and prothrombin. Maintaining pregnant women who actively experience migraines with aura under close observation may be of some value in achieving better outcomes. Women who experience migraines after starting hormonal contraception are at a higher risk of experiencing strokes and stand to benefit from being switched to non-hormonal methods. In this article, we discuss the mechanisms linking migraines and strokes, briefly discuss the pathogenesis, and explore the risk factors contributing to the association therein. In addition, we examine the relationship between migraines and ischemic strokes, as well as hemorrhagic strokes, and review management considerations.

## Introduction and background

Chronic, recurrent headaches, particularly migraines, have become one of the leading causes of disabilities globally [1]. Recent studies estimate that 12% of the US population meets the clinical criteria for chronic or episodic migraines [2]. The National Health Interview Survey published in 2018 demonstrated a 15% prevalence of self-reported migraines. The same survey showed that 10% of all males and 20% of all females suffered from this ailment [3]. Symptomatic migraines are defined as a neurologic disorder characterized by debilitating headaches, which may often be accompanied by sensory as well as motor function deficits [4]. When the somatosensory disturbance presents immediately before the headache, it is called a migraine with aura, although migraines can also present without aura [5]. Although the causes of migraines are multifactorial, it is widely accepted that circulating pro-inflammatory agents and free radicals contribute largely by triggering the trigeminovascular system [6,7]. The following five features render a headache most likely a migraine: pulsatile (pounding/throbbing), lasting for one day (although it can last up to 72 hours if untreated), unilateral, accompanied by nausea, and debilitating in intensity [8]. The International Headache Society has its own criteria for migraine diagnosis, and although it boasts of an incredible specificity, its sensitivity remains under 50% [8,9]. Management includes identifying and eliminating dietary triggers such as caffeine, monosodium glutamate, and artificial sweeteners, as well as treating the headache pharmacologically with acetaminophen, aspirin, or triptans in more severe cases [10-14]. Propranolol, divalproate, and topiramate have been used successfully for the preventive management of migraines [8]. As prevalent as migraines are, recent studies have shown migraines to be associated with stroke which is among the leading causes of death worldwide, trailing only a few positions behind ischemic heart disease, which together caused a total of 15.2 million deaths worldwide in 2015 [15]. This unusual correlation has presented a challenging dilemma in the field of neurology making it imperative to formulate diagnostic and therapeutic guidelines. This review aims to explore correlations between mechanisms, risk factors, and pathways shared between these two neurovascular diseases with a brief focus on the diagnostic and management options.

## Review

Although migraines are widely believed to be caused by an eventual triggering of the trigeminovascular system that leads to vasodilation of the meningeal vessels [16], more recent studies have shown that this event is only secondary to a stimulus, and the origin of the pain is within the brain regions [17]. Some of the regions implicated in the pathogenesis of migraine pain include sensory and central nerve terminals in the meninges and sensory nuclei of the brainstem [18], the cingulate cortex [19], dorsal rostral brainstem [20], the red nucleus and substantia nigra [21], the dorsolateral pons [22], and the hypothalamus [23]. With their pathogenesis deeply embedded in the neural, vascular, and structural aspects of the brain, it is no surprise that they relate in so many ways to strokes.

Mechanisms linking migraines to strokes

There are several theories regarding what might contribute to hemorrhagic strokes in patients with migraines. First, the circulating number of progenitor endothelial cells is decreased in migraineurs [24]. This, along with an alteration in the vessel wall structure seen in patients acutely reporting migraines, can set up a favorable environment for hemorrhagic strokes [25]. Second, vascular pathologies such as platelet dysfunction [26], hypertension, and high cholesterol profiles are all risk factors that contribute to migraines as well as hemorrhagic and ischemic strokes. Scher et al. studied a cohort of 5,755 patients aged 20-65 years with an equal gender distribution. The patients were compared with a control group of 5,135 individuals with no history of migraines. The study showed that migraineurs with aura were more likely to have higher cholesterol profiles, with total cholesterol greater than or equal to 240 mg/dL (odds ratio [OR]: 1.43; 95% confidence interval [CI]: 0.97-2.1). The study also showed that migraineurs were more likely to have higher blood pressures, with a systolic blood pressure of >140 mmHg and diastolic blood pressure of >90 mmHg (OR: 1.76; 95% CI: 1.04-3.0). This shows that patients who have migraines with aura have a higher cardiovascular risk profile that may contribute to their increased risk of stroke [27].

Another mechanism relates to a shared risk factor, namely, non-steroidal anti-inflammatory drugs (NSAIDs). Among individuals who use NSAIDs for migraine pain control, having 10 or more headache days per month makes NSAIDs a contributing factor to migraines rather than preventing progression [28]. In addition, NSAIDs are associated with a high risk of stroke, particularly ibuprofen, making them a possible contributory factor to hemorrhagic strokes in patients with migraines [29]. Mechanisms linking migraines to ischemic strokes have been hypothesized equally heavily. One possible mechanism is progressive hypoperfusion and reduction in cerebral blood flow that occurs during migraine [30]. The mechanism by which this hypoperfusion occurs is called “spreading depression” [31] which is a marked reduction in potential generating activity within neuronal membranes in the gray matter that advances across the cortex at a rate similar to the one seen with the progression of symptoms in migraines [32,33].

Another possible mechanism of cerebrovascular hypoperfusion is vasospasm [34]. Several case reports have established this link. Fujita et al. reported vasospasm in the left middle and posterior cerebral arteries in a 10-year-old girl with migraines [35]. In another case report, Marshall et al. reported a woman who presented with an exacerbation of her migraine headaches followed by a series of strokes that lead to her death. The 57-year-old had no vascular or hematologic risk factors, nor was she on any drugs [36]. In several other reports, arterial vasoconstriction has been noted even after ruling out any potential mimicry [37,38].

The association between migraines and strokes is further supported by increased concentration and activation of several intravascular procoagulant factors in migraineurs. One of these is the antiphospholipid antibody [39] which increases hypercoagulability by increasing the affinity of phospholipid complexes to other phospholipids, as well as cell surface. In addition, they can trigger platelet activation [40]. Another procoagulative factor found in increased concentrations among migraineurs is homocysteine, specifically the methylenetetrahydrofolate reductase (MTHFR) C677T genotype, which is associated with an increased risk of stroke. Scher et al. found an OR of 2.05 (95% CI: 1.2-3.4) for stroke in patients with MTHFR C674T homozygosity when compared to the wildtype genotype, independent of all other genotypes [41].

Another procoagulative agent implicated in migraines related to stroke is the von Willebrand factor (vWF). Tietjen et al. reported in 2001 that migraine patients with prior strokes had 170% higher concentrations of vWF compared to 106% in frequency-matched controls, as well as activation of vWF at 162% versus 108% in controls [42]. In addition to vWF, studies have also reported increased concentrations of endothelin-1, a potent vasoconstrictor [43], as well as prothrombin 1.2, the cleavage product of prothrombin. Prothrombin 1.2 is a sensitive and specific marker of ongoing thrombosis and is speculated to be an indicator of increased coagulability. In another study, Hering-Hanit et al. reported significantly increased levels of prothrombin 1.2 in patients who had migraines with aura, but not so much in migraines without aura [44].

**Figure 1:**
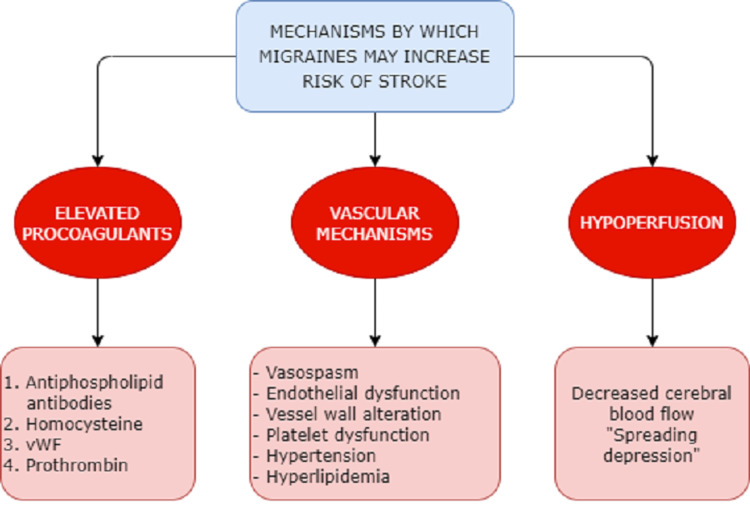
Mechanisms contributing to the link between migraines and strokes. vWF: von Willebrand factor

Risk factors with gender as a predominantly prevalent element

With several studies linking migraines to strokes and clearly hypothesized mechanisms, we would be remiss not to discuss risk factors that may be shared by and contribute to each other’s etiologies.

Gender plays a significant role as a risk factor in both migraines and strokes. At younger ages, women have an equal or higher risk of stroke as men [45]. Asplund et al. studied a cohort of 93,695 women across 18 European countries and analyzed 3,142 stroke events. They reported that the risk of stroke increased by 10% per year in women and 9% in men [46]. Similarly, the prevalence of migraine is three times higher in women than in men [47]. This may be one of the reasons why the risk of stroke in women with migraines appears to be considerably higher in other studies, particularly in women younger than 45 [47-49]. It is estimated that up to 37% of women will experience migraines at some point in their life [50]. The lifetime risk of stroke in women is one in five [51].

Other factors can also contribute to this gender disparity such as pregnancy. Migraine as a risk factor for pregnancy-related stroke was assigned an odds ratio of 16.9 (95% CI: 9.7-29.5) in a nationwide inpatient population study conducted in 2005 [52]. The incidence of stroke in women of childbearing age is approximately 1 in 10,000. This increases to nearly 3.5 per 10,000 during pregnancy [53]. This could be due to several physiologic changes experienced during pregnancy, ranging from venous stasis to edema to the hypercoagulability experienced during gestation [54]. Active migraines with aura during pregnancy can pose a potential risk for adverse vascular outcomes, particularly strokes of ischemic etiology, and migraineurs with known risk factors for stroke benefit from high-risk pregnancy monitoring [55,56].

Another factor contributing to gender disparity may be oral contraceptive pill (OCP) use. A World Health Organization (WHO) collaborative study conducted in 1999 showed that women who used OCPs and experienced migraines were eight times more likely to suffer from an ischemic stroke when compared to women with either one of the risk factors by itself. The same women were 16 times more likely to experience a stroke when compared to those who had neither risk factor [57].

An unlikely risk factor linking migraines to strokes is patent foramen ovale (PFO). When the fetal structure fails to close, blood continues to pass from the right atrium to the left, bypassing pulmonary circulation. This allows the mixing of arterial and venous blood, which may result in paradoxical emboli breaking off from venous thrombi and causing ischemic strokes [58]. Several studies have shown a higher prevalence of migraines among individuals with PFOs. One systematic review identified 18 articles to study the strength of association between migraines and PFO. The study reported an odds ratio of 5.13 (95% CI: 4.67, 5.59) for migraines of all types. The study also reported that the closure of PFO affected migraine patterns positively [59].

Smoking is another risk factor that increases the likelihood of migraineurs experiencing strokes at some point in their life, particularly in the elderly [47]. There is evidence suggesting that smoking can cause migraine attacks and that the prevalence of smoking is 33% higher in migraineurs, with a positive relationship between migraine attacks and the number of cigarettes smoked [60]. A meta-analysis conducted to assess the risk of stroke among smokers found a pooled OR of 1.61 (95% CI: 1.34-1.93) compared to non-smokers. The same analysis found an OR of 1.92 (95% CI: 1.49-2.48) among current smokers and 1.30 (95% CI 0.93-1.81) among former smokers. Even passive smoking increased the risk by 45% (OR: 1.45; 95% CI: 1.0-2.11) [61]. One of the mechanisms by which passive smoking increases the risk of stroke is the oxidation of low-density lipoprotein cholesterol [62]. Another mechanism is atherosclerosis of the carotid arteries, even among individuals without additional cardiovascular risk factors [63].

Another factor contributing to the increased risk of stroke in migraineurs is migraine treatments [64]. Ergotamine, a popular class of drugs used in the treatment of migraines, has been available since 1926, with dihydroergotamine introduced 20 years later [65]. Ergotamines act on 5HT1b/d receptors on serotonergic neurons, inhibiting neuronal inflammation but causing arterial vasoconstriction in the process [66]. This mechanism can explain why patients with pre-existing cardiovascular disease are at an increased risk for cardiovascular and cerebral ischemic episodes [67]. On the other hand, modern migraine pharmacotherapeutic agents such as fremanezumab, eptinezumab, and galcanezumab, all belonging to a class of drugs known as calcitonin gene-related peptide receptor antagonists, cause no side effects pertaining to vasoconstriction and might be better agents for the treatment of migraines among patients with a high cardiovascular risk profile [68].

Lastly, migraines carry a genetic component which is demonstrated by their high heritability rates. Twin studies determined a heritability rate of 65% in patients with migraines with aura and 52% in patients with migraines without aura [69]. If migraine and stroke have a partly shared vascular pathogenesis as described earlier, and the high heritability of migraines, it is highly likely that they also share genetic risk factors. In 2015, Malik et al. analyzed several genome-wide association studies for both migraines and ischemic stroke and determined a combined contribution to both pathologies via numerous genetic variants at common loci [70]. The strongest genetic overlap with all migraine types was found in large artery and cardioembolic strokes. When loci previously shown to reach genome-wide significance for association with migraines were analyzed, several variants were reported to be associated with ischemic strokes. One such variant was 9p21, a major locus for large artery strokes [70-72].

Migraine and the risk of ischemic stroke

The risk of stroke is doubled in patients with migraines compared to patients who are migraine-free, and this is especially prevalent in patients who suffer from migraines with aura [47]. In 2017, Lantz et al. conducted a twin cohort study in a Swedish population of 54,404 subjects. Of those, 8,635 twins reported migraine headaches, with 5,082 reporting migraines without auras and 3,553 reporting migraines with auras. Over the course of 12 years, 1,297 incidents of stroke were observed among this population. The researchers discovered that the hazard ratio (HR) for stroke related to migraine with aura was 1.09 (95% CI: 0.81-1.46; P = 0.59) (Table 1) [73]. In contrast, a multi-center prospective cohort study conducted in the United States by Rambarat et al. in 2017 reported a twofold increase in the risk of stroke (HR: 2.33; 95% CI: 1.16-4.68) in women patients who reported a migraine compared to those who did not. This study followed 224 women over the course of 6.5 years (Table 1) [74].

A much larger study corroborated the above findings where 115,541 women aged 25-42 years were followed for 22 years. Kurth et al. reported that the risk of stroke was significantly higher in women reporting migraines (HR: 1.62; 95% CI: 1.37-1.92) compared to those without migraines (Table 1) [75]. In addition to women being at an increased risk, as demonstrated in the previous two studies, another interesting confounder of stroke risk in older migraine patients (mean age of 68 ± 9 years) was reported by Monteith et al. in 2015 when analyzing data of 337 patients who reported migraines from a total of 1,292 stroke patients followed for 11 years. The study found that smoking increased the risk of stroke in people with migraines nearly threefold (HR: 3.17; 95% CI: 1.13-8.85) (Table 1) [76]. The association of migraine with stroke is not limited to the adult and adolescent populations only. Gelfland et al. reported that among 1.56 million pediatric (aged 2-17 years) patients in Kaiser Permanente Northern California, 88,164 reported migraines over the course of 10 years (1997-2007). Only eight of those children with migraines had strokes, whereas stroke occurred in 80 children without headaches. A post hoc analysis of the same population in adolescence (aged 12-17 years) showed a significant increase in the risk of ischemic stroke among those with migraine, with an incident rate of 3.4 (95% CI: 1.2-9.5) which supports the aforementioned studies showing two to threefold increase in the same (Table 1) [77]. The aforementioned studies and pertaining information is presented in Table 1.

**Table 1:** Summary of studies exploring the association between migraines and strokes. HR: hazard ratio; CI: confidence interval

Study (reference)	Design	Migraine cases	Cohort size	Population	Follow-up years	Findings
Lantz et al. (2017) [73]	Twin retrospective cohort study	8,635	54,404	Twins in Sweden	12	HR: 1.09 95% CI: 0.81-1.46
Rambarat et al. (2017) [74]	Prospective cohort study	224	917	Women in the United States	6.5	HR: 2.33 95% CI: 1.16-4.68
Kurth et al. (2016) [75]	Retrospective cohort study	17,531	115,541	Women aged 25-42 years	22	HR: 1.62 95% CI: 1.37-1.92
Monteith et al. (2015) [76]	Prospective cohort	337	3,298	Smokers aged 68 ± 9 years	11	HR: 3.17 95% CI: 1.13-8.85
Gelfland et al. (2015) [77]	Retrospective cohort study	88,164	1,566,952	Children aged 2-17 years	6.5	HR: 3.4 95% CI: 1.2-9.5

Migraine with aura and hemorrhagic stroke

Although ischemic stroke is more prevalent, hemorrhagic stroke is responsible for the most morbidity and mortality [78]. Between 1990 and 2013, the number of deaths from ischemic strokes was approximately 0.4 million, whereas the number of deaths from hemorrhagic strokes was well over double at 1.0 million [79]. Therefore, it is imperative that any review aiming to determine the associations between migraines and strokes considers hemorrhagic strokes separately. One prospective cohort study conducted in 2010 found that women who reported active migraine without aura had no increased risk of hemorrhagic stroke. Kurth et al. studied a cohort of 27,860 women with no history of stroke at baseline but reported their migraine status as well as their aura status. Over a 13.6-year follow-up, 5,130 of those women reported migraines, 60% of which were without aura. Although there was no increased risk of hemorrhagic stroke in women reporting any history of migraine compared to those reporting no migraines at all (HR: 0.98; 95% CI: 0.56-1.71), women with active migraines with aura exhibited a hazard ratio of 2.25 (95% CI: 1.11-4.54) [80]. This finding directly contrasted a previous study by the same authors in 2004. Whereas the cohort was larger at 39,754 women, the cases of stroke were considerably lower at only 385. The authors concluded that migraines were not associated with strokes of any sort, ischemic or hemorrhagic [81].

The abovementioned findings are also supported by another study conducted by Gaist et al. in 2014. The study obtained data of 3,137 stroke patients and compared them with a control group involving 10,000 stroke-free patients to calculate the risk of hemorrhagic strokes using unconditional logistic regression models. The study found an OR for hemorrhagic events among migraineurs to be 1.2 (95% CI: 0.9-1.5). When the results were stratified by migraine type, that is, migraine with or without aura, the OR in women who experienced migraines with aura was 1.7 (95% CI: 0.6-2.3) [82].

In contrast to all the abovementioned studies, only one study reported an association between migraine without aura and hemorrhagic stroke, in addition to migraine with aura and hemorrhagic stroke. Kuo et al. followed a cohort of 20,925 people with a diagnosis of migraine in Taiwan for two years. A non-migraine group of 104,625 age and sex-matched subjects without migraines was used as the control group. The study reported an HR for developing hemorrhagic stroke in the migraine group to be 2.22 compared to patients without migraines (95% CI: 1.78-2.77). The migraine without aura subgroup alone had an HR of 2.01 (95% CI: 1.25-3.25) [83].

Treatment considerations

As evident from this review, it is advisable to pay special attention to migraines in patients with certain risk factors. Special consideration should be given to women, particularly women with aura. Pregnancy in women who actively experience migraines with aura is associated with adverse outcomes and can benefit from active monitoring [55,56]. The risk of stroke associated with migraines is higher in women who are on hormonal contraception, and careful consideration is required when starting female migraineurs with aura on such drugs [84]. Women who develop migraines after starting contraception may benefit from switching to a non-hormonal contraceptive [50,85]. The risk of stroke remains high in PFO; a clinical trial is currently underway to determine if migraineurs stand to benefit from the transcatheter closure of the PFO [86].

Limitations

This review article has several limitations. First, migraines and strokes can both mimic and present with overlapping clinical features in various stages of their course. Thus, a diagnostic misclassification can be a likely source of skewed results. Second, both strokes and migraines have several subtypes attributing to varying degrees of clinical symptoms. Silent strokes and silent migraines are both widely studied phenomena, and it is difficult to analyze when searching for an association between the two. Finally, regarding genetic overlap and heritability, the power of the studies analyzing these facets is proportional to the overall correlation between the effects on the two traits under investigation. In genome-wide association studies particularly, the association is stronger in migraines without aura, and no loci have been identified yet for migraines with aura.

## Conclusions

This review highlights previous research and studies that explore the relationship between migraines and stroke. Based on the aforementioned evidence, migraines, particularly migraines with aura, should be considered an important risk factor for ischemic stroke. Additionally, hemorrhagic strokes are associated with migraines without aura. Women are particularly at an increased risk for migraine-associated strokes. Previous studies have recommended switching to non-hormonal contraception in cases where migraines begin after starting hormonal contraception to lower the risk of stroke in this subset of patients. The higher prevalence of migraines among women, as well as the increase in vascular risk factors associated with pregnancies and contraceptive use, can be a contributing factor to the association between migraine and stroke. Patients with PFOs are at an increased risk for stroke due to the mechanisms involved therein; however, there is little evidence to support migraine relief from PFO closure. Multiple other possible mechanisms warrant a well-designed investigation. More longitudinal studies, particularly in a sizable subset of young women who experience migraines with aura, will be useful in the future to determine if treating migraine lowers the risk of stroke in premenopausal women.
